# Restoration of Two-Photon Ca^2+^ Imaging Data Through Model Blind Spatiotemporal Filtering

**DOI:** 10.3389/fnins.2021.630250

**Published:** 2021-04-16

**Authors:** Liyong Luo, Yuanxu Xu, Junxia Pan, Meng Wang, Jiangheng Guan, Shanshan Liang, Yurong Li, Hongbo Jia, Xiaowei Chen, Xingyi Li, Chunqing Zhang, Xiang Liao

**Affiliations:** ^1^Brain Research Center and State Key Laboratory of Trauma, Burns, and Combined Injury, Third Military Medical University, Chongqing, China; ^2^Department of Patient Management, Fifth Medical Center, Chinese PLA General Hospital, Beijing, China; ^3^Brain Research Instrument Innovation Center, Suzhou Institute of Biomedical Engineering and Technology, Chinese Academy of Sciences, Suzhou, China; ^4^Center for Neurointelligence, School of Medicine, Chongqing University, Chongqing, China; ^5^Department of Neurosurgery, Xinqiao Hospital, Third Military Medical University, Chongqing, China

**Keywords:** image restoration, model blind learning, spatio-temporal processing, residual convolutional network, machine learning, two-photon Ca^2+^ imaging

## Abstract

Two-photon Ca^2+^ imaging is a leading technique for recording neuronal activities *in vivo* with cellular or subcellular resolution. However, during experiments, the images often suffer from corruption due to complex noises. Therefore, the analysis of Ca^2+^ imaging data requires preprocessing steps, such as denoising, to extract biologically relevant information. We present an approach that facilitates imaging data restoration through image denoising performed by a neural network combining spatiotemporal filtering and model blind learning. Tests with synthetic and real two-photon Ca^2+^ imaging datasets demonstrate that the proposed approach enables efficient restoration of imaging data. In addition, we demonstrate that the proposed approach outperforms the current state-of-the-art methods by evaluating the qualities of the denoising performance of the models quantitatively. Therefore, our method provides an invaluable tool for denoising two-photon Ca^2+^ imaging data by model blind spatiotemporal processing.

## Introduction

Understanding how brain information processing is implemented during cognitive tasks requires the interpretation of neuronal activities at multiple scales (Romo and De Lafuente, 2013; Guo et al., 2014). Monitoring neuronal activities at high resolution is crucial for the investigation of brain functions (Grewe et al., 2010; Huber et al., 2012; Tischbirek et al., 2019). Two-photon microscopy (Denk et al., 1994; Helmchen and Denk, 2005; Grienberger and Konnerth, 2012) is a powerful and versatile neuroimaging technique for recording neuronal dynamics using Ca^2+^-dependent fluorescent indicators and can produce neuronal recordings as video data. The optical nature of two-photon Ca^2+^ imaging allows precise observation of the spatial localization of cells, thus facilitating the analysis of the underlying relationship between cell activity and its location (Wang M. et al., 2020). This further helps to guide patch pipettes when performing electrophysiology recordings (Ding et al., 2017) and in differentiating between cell types (Chen J. L. et al., 2013). Moreover, two-photon microscopy enables us to monitor neuronal activities at different scales, ranging from hundreds, or even thousands, of neurons (Stosiek et al., 2003; Svoboda and Yasuda, 2006; Peron et al., 2015) to single dendrites (Jia et al., 2010) and spines (Chen et al., 2011; Chen X. et al., 2013) of individual neurons. Recently, remarkable advances in two-photon microscopy have led to the simultaneous recording of several brain areas (Yang et al., 2019) and even the tracking of neuronal activity over a period of weeks (Peters et al., 2014; Li et al., 2018).

However, two-photon imaging systems are inherently noisy because the number of photons reaching the microscopic detector is relatively small (Morris et al., 2015). This measurement noise is composed of complex components, including Poisson noise for the photons reaching the photodetector and added white Gaussian noise for thermal fluctuations (Zhang et al., 2019). Although it is possible to obtain cleaner data by increasing the imaging time, this is accompanied by an increased risk of sample damage. To date, the physical limits of two-photon microscopy (imaging depth, imaging speed, spatial resolution, and image quality, etc.) are still difficult to overcome by simply optimizing the microscopy hardware. Therefore, developing computational tools to improve image quality is becoming increasingly important for microscopy data processing. Denoising, deblurring, and single-image super-resolution algorithms are representative image restoration approaches that enable the recovery of important biological information that has been subject to complex corruptions, thus pushing the limits of two-photon microscopy (Weigert et al., 2018). Signal denoising, which aims to retrieve the true signal from noisy images, aims to become a crucial module in the analysis arsenal of two-photon imaging data (Guan et al., 2018; Belthangady and Royer, 2019). Therefore, developing a denoising algorithm for effectively restoring two-photon Ca^2+^ imaging data can significantly help data processing in neuroscience research.

Reconstructing the true signals from noisy or incomplete measurements is an important and enduring challenge in the signal processing field. Many previous studies have suggested solutions for restoring images. These algorithms are traditionally based on statistical models, for example, Markov random fields (Roth and Black, 2005), sparse representations (Elad and Aharon, 2006), and nonlocal means (Buades et al., 2005). Block matching and 3D filtering (BM3D) is a popular state-of-the-art tool for denoising images (Dabov et al., 2007). Deep learning is a recent fast-developing field; artificial neural networks with multiple layers are trained using a large amount of labeled data, thus learning a complex mapping process that translates training data to produce the desired output (Lecun et al., 2015). In neuroscience, deep learning algorithms have been applied successfully to various tasks, for example, the automatic reconstruction of neurons from volume electron microscopy data (Januszewski et al., 2018), cell segmentation (Falk et al., 2019), fluorescence label prediction from unlabeled images (Christiansen et al., 2018), and accelerating super-resolution localization microscopy (Ouyang et al., 2018). Recent advances in using deep learning methods to restore microscopy images have shown significant quality improvements by learning to transform corrupted measurements to clean data (Weigert et al., 2018); deep learning algorithms outperform traditional statistical modeling methods, which require explicit knowledge of data corruptions. To our knowledge, while many methods have been used for denoising single images, few methods have been developed for denoising video data, such as functional Ca^2+^ imaging data. For video denoising, it is important to note that the consecutive frames in video data are, in general, highly correlated but are susceptible to motion-induced artifacts. Block matching and 4D filtering (BM4D) is an extension of the BM3D single-image denoising method and is a state-of-the-art video denoising tool (Maggioni et al., 2012). It searches similar patches in both spatial and temporal dimensions, thereby increasing the computational cost drastically. Therefore, a spatiotemporal or 3D convolutional network architecture that combines spatial and temporal filtering will be more robust to artifacts caused by brightness changes and object motion (Claus and Van Gemert, 2019; Wang C. et al., 2020). The spatiotemporal architecture can be superior to the network models using only spatial information, and it has already shown some promising results for the analyses of video (Soltanian-Zadeh et al., 2019) and volumetric (Kamnitsas et al., 2017) neural data.

In this work, we propose to use a model blind learning approach that combines spatial and temporal information to restore two-photon Ca^2+^ imaging data. Unlike traditional methods, this method does not require ground truth imaging data with a high signal-to-noise ratio (SNR). The complex processing required to denoise raw imaging data is implemented *via* end-to-end learning, based on observing noisy data only. In addition, spatiotemporal information is used for the network model to tackle temporal inconsistencies and recover true signals in both the spatial and the temporal dimensions. Hence, the mapping of the network model is represented as a model blind spatiotemporal filtering, which is learned, with a high SNR, by the output imaging data. The performance of the proposed network was first validated by using synthetically generated noisy two-photon imaging data and then using real raw two-photon imaging data. Subsequent quantitative assessments of the restored two-photon imaging data demonstrate that both the spatial and temporal signal qualities exhibit significant improvements in comparison with the methods reported previously. Thus, the tests performed with the proposed network show that it provides efficient denoising for two-photon Ca^2+^ imaging data *via* a model blind spatiotemporal processing, and thereby offers a solution to facilitate the restoration of massive imaging datasets in neuroscience research.

## Materials and Methods

### Data Acquisition

For the experiments, C57BL/6J mice (2–3 months old) were provided by the Laboratory Animal Center at the Third Military Medical University. All procedures were carried out in accordance with protocols approved by the Third Military Medical University Animal Care and Use Committee.

For the two-photon Ca^2+^ imaging experiments, recoding was performed in the primary auditory cortex of the mice (Li et al., 2017a, b; Wang et al., 2018). The mice were anesthetized using isoflurane and their body temperature was maintained at 37.5°C. After a local lidocaine injection, the skin and muscles over the targeted cortex region were removed. A prefabricated plastic chamber was glued to the skull, and a small craniotomy (∼4 mm^2^) was used to expose the auditory cortex. The craniotomy was filled with 1.5% low-melting-point agarose, and the chamber was perfused with normal artificial cerebral spinal fluid. The dye Cal-520 AM was injected into the auditory cortex of the mice, and two-photon Ca^2+^ imaging was performed 2 h after dye injection.

*In vivo* two-photon Ca^2+^ imaging was performed using a custom-built two-photon microscope system (LotosScan, Suzhou Institute of Biomedical Engineering and Technology, China) (Jia et al., 2010, 2014). In the mice cortices, two-photon excitation light was delivered with a mode-locked Ti:Sa laser (Mai-Tai DeepSee, Spectra Physics, Santa Clara, CA, United States). The laser was focused by a 40×/0.8 NA water-immersion objective lens (Nikon) onto the brain tissue. The excitation wavelength was set to 920 nm for all Ca^2+^ imaging experiments. For different imaging depths, the average laser power was adjusted in the range of 30 to 120 mW. The imaging data were acquired at a spatial resolution of 500 × 500 pixels and a sampling rate of 40 Hz. The imaging data were then analyzed offline.

### Model Blind Spatiotemporal Filtering Network

**FIGURE 1:**
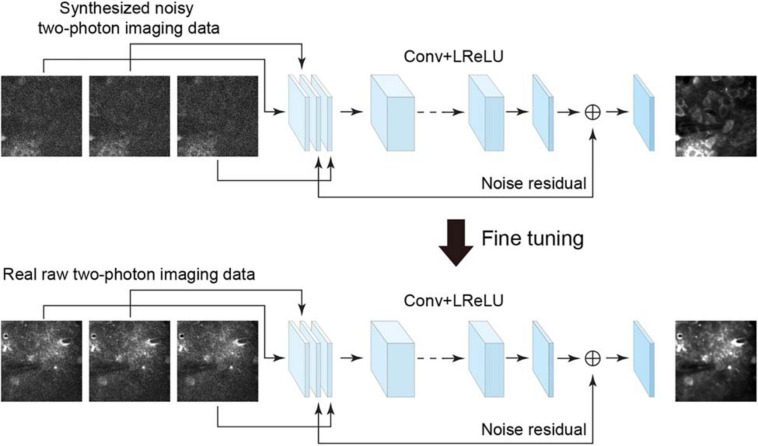
Schematic illustration of the neural network for model blind spatiotemporal denoising. The neural network trained using the synthesized noisy imaging dataset can be fine-tuned to analyze the real two-photon imaging dataset by noise-to-noise learning. Conv, 2D convolutional layer; LReLU, leaky rectified linear unit.

To denoise the acquired raw imaging data, an efficient approach is to use a residual learning-based convolutional neural network, e.g., denoising convolutional neural network (DnCNN) (Zhang et al., 2017), which showed remarkable effectiveness when dealing with several image denoising tasks using the same architecture. However, the DnCNN does not use temporal information and processes each image independently. To combine the spatial and temporal information for residual learning and improving denoising quality, we used the neural network architecture implemented as the temporal denoising part of the video denoising neural network (ViDeNN) (Claus and Van Gemert, 2019) to perform spatiotemporal filtering. The network for spatiotemporal denoising was constructed based on a residual structure (Figure 1), for which the network input was three consecutively stacked frames, which has been shown to be efficient in previous studies (Su et al., 2017; Claus and Van Gemert, 2019). The network combined the spatiotemporal information from these three imaging data frames and estimated the residual noise for the central frame, and it utilized motion information among these successive frames to capture temporal inconsistencies. The network model comprises 20 layers, with the first layer comprising 128 filters and the remaining layers 64 filters. The size of each filter was set to 3 × 3 × *c*, where *c* is the number of image channels. Although the input has multiple frames, the network does not need an additional layer for processing the consecutive frames. The least square error (i.e., L2-norm) between the desired residual values and the estimated values was calculated as the loss function. The leaky rectified linear unit (LReLU) activation function was used in the network model. Therefore, by using the temporal denoising model of ViDeNN as the backbone for the spatiotemporal filtering network, the model architecture enables the network to handle different types of noises in the imaging data.

In some recent studies, for example, Lehtinen et al. (2018), the authors have shown remarkable results indicating that a denoising model can be trained directly from certain types of noisy images being presented, which is called noise-to-noise learning, with the hypothesis that the noisy images are independent observations from the same clean data. For this type of blind learning framework, clean data are not a necessity for training the network model. This enables a denoising network model to learn from noisy data only when clean data cannot be obtained easily. Based on this noise-to-noise learning strategy and also inspired by a recent study of denoising video data *via* a frame-to-frame fine-tuning procedure (Ehret et al., 2019), we adapted the spatiotemporal filtering network by incorporating an extended noise-to-noise learning framework. We extended the noise-to-noise learning strategy from a training network with single images to a training network with consecutive frames (image stack) in imaging data. To perform model blind spatiotemporal filtering, we carried out the following training procedures: (i) we initially trained the spatiotemporal filtering network model with two-photon imaging data for which noise had been added synthetically, and (ii) we applied this extended noise-to-noise learning to real raw imaging data frames to fine-tune the initially trained network. The fine-tuning stage consisted of initializing the network with loaded pretrained network weights and retraining the whole network by updating the pretrained weights on real raw two-photon imaging dataset only. Thus, we adapted the spatiotemporal denoising model (temporal denoising part of ViDeNN) to a model blind spatiotemporal filtering network by using our extended noise-to-noise training strategy, and hence, the network model can be optimized and enabled to handle unknown noise components in real imaging data.

To train the neural network, we used the adaptive moment estimation (Adam) (Su et al., 2017; Claus and Van Gemert, 2019) optimizer to minimize the expected error between the model output and the target data. The learning rate was set to a constant value of 0.0001 over 50 epochs for initial training and 10 epochs for fine-tuning.

### Dataset for Training and Validation

For the initial training of the neural network, the training data were synthesized with Poisson and Gaussian noise components, as presented in previous studies (Foi et al., 2008; Makitalo and Foi, 2012; Zhang et al., 2019). The Poisson noise and Gaussian noise account for the signal-dependent and signal-independent uncertainties, respectively. For the noise model, we let *m*_i_, where *i* = 1, 2, …*N*, represent the two-photon microscopy data sample,

(1) $$m_{i}=t_{i}+n\,s_{\text{p}}\,\left(t_{i}\right)+n\,s_{\text{g}}$$

where *t*_i_ is the ground truth; *ns*_p_ is the Poisson noise, which is a function of *t*_i_; and *ns*_g_ is the zero-mean Gaussian noise. The parameters for generating synthetic images were determined by comparing the peak signal-to-noise ratio (PSNR) and structural similarity (SSIM) metrics, so that the synthetic images are close to the real raw two-photon images.

The training of the network model for restoring imaging data requires pairs of corrupted inputs and clean targets. However, obtaining ground truth clean data is difficult in experiments. As suggested by Zhang et al. (2019), image averaging is equivalent to sampling with a higher SNR; therefore, it is an effective approach to obtain the approximated ground truth images. Hence, we used image averaging to estimate the reference of the ground truth for constructing denoising dataset, and we obtained the reference by averaging 100 imaging data frames captured using the same field of view (FOV). We added Poisson and Gaussian noises to a reference image to construct the synthetic noisy imaging dataset and prepared them as sequences of three image frames with Poisson and Gaussian noises of the same magnitudes. For each one of those sequences, we stacked three frames as the network input and the reference image of the ground truth (three replications) as the network target (*n* = 1,200 image pairs).

After the initial training of the network, we collected real raw imaging data for the model blind learning. We treated consecutive imaging frames as independent noisy observations of the same clean data and divided the imaging data into sequences of four consecutive frames. For each one of those sequences, we used the first three consecutive frames as the network input and the fourth frame (three replications) as the network target (*n* = 900 image pairs).

### Evaluation

For the functional imaging dataset, for which imaging was performed for the same FOV, we were able to obtain an estimated reference of the ground truth. Therefore, we adopted two popular image quality metrics, that is, the PSNR and the SSIM index, to evaluate network performance. The quality relationship between the reference and noisy images and the restored images were calculated, respectively.

In the experiment of morphological imaging, that is, imaging performed for different FOVs (i.e., z-stack), it is infeasible to average many imaging frames from one FOV to estimate the reference of ground truth data. In the case of testing the image quality without reference image, PSNR, and SSIM metrics are not applicable, and hence, a no-reference image quality evaluation algorithm was adopted to assess this type of imaging data, namely, the blind image quality index (BIQI) (Moorthy and Bovik, 2010). The BIQI ranges between 0 and 100, with a smaller BIQI score indicating better image quality.

In addition to evaluating the denoising performance in the spatial domain, we also quantified the SNR for the neuronal signals in the time domain. For an imaging FOV, we identified neurons manually and marked them as regions of interest (ROIs). The fluorescence trace (*f*) of individual neurons over time was calculated by averaging the corresponding pixel values within each specified ROI. Changes in the Ca^2+^ signal (i.e., relative fluorescence) were calculated as Δ*f*/*f* = (*f* - *f*_0_)/*f*_0_, where the baseline level of fluorescence *f*_0_ was taken as the 25th percentile of the fluorescence trace. The peak amplitude of the Ca^2+^ transient was calculated as the difference between the baseline level (average Δ*f*/*f* of 2 s) and the peak level (peak-centered average Δ*f*/*f* of 75 ms). The SNR for the Ca^2+^ transient was defined as the ratio of the peak amplitude of the Ca^2+^ transient to the standard deviation (*SD*) of the baseline fluctuation (Tada et al., 2014):

(2) $$S\,N\,R=\frac{P\,e\,a\,k-m\,e\,a\,n\,\left(B\,a\,s\,e\,l\,i\,n\,e\right)}{S\,D\,\left(B\,a\,s\,e\,l\,i\,n\,e\right)}$$

## Results

**FIGURE 2:**
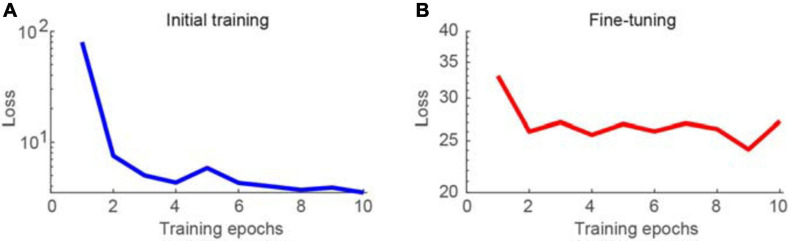
Learning curves for training the network model. **(A)** Training loss as a function of epoch for initial training of the network, visualizing for the first 10 epochs. **(B)** Training loss as a function of epoch for fine-tuning of the network.

First, the proposed network was trained using synthesized Poisson and Gaussian noise data, before being fine-tuned using raw noisy imaging data. The training process was conducted using the parameters described in the section “Materials and Methods.” The learning curves for the initial training and fine-tuning of the network are shown in Figures 2A,B, respectively. The training loss was decreased dramatically for the initial training stage, particularly at the second epoch, as the loss could be greatly reduced by learning to map noisy observations to clean signals. When switching the training data from pairs of noisy images and a clean target to pairs of noisy images, the loss changed to be much larger. In contrast, the training loss was not decreased much and continued to be large when performing noise-to-noise learning for the fine-tuning stage, which is consistent with the training results in a previous work (Lehtinen et al., 2018). In our tests, we stacked three consecutive frames for denoising and obtained a clean version of the middle frame. The calculation time was 70 ms for each image of 500 × 500 pixels using an Intel i9 7980XE 2.9 GHz CPU, with 128 GB RAM, and NVIDIA’s GTX 1080 Ti GPU. In the figures, the brightness of the representative two-photon images was adjusted for better visualization.

### Experimental Validation Using Synthetic and Raw Imaging Data

To first perform an experimental validation of the proposed method, we evaluated the denoising results on the imaging data with synthetically added noise. The synthetic noisy imaging data were generated using a Poisson and Gaussian noise model as described in the section *“*Dataset for Training and Validation.” Poisson noise with a magnitude of 1 and Gaussian noise with standard deviation sampled randomly from (0, 0.05) were added to the reference images to generate training and testing images. The magnitudes for Poisson noise and Gaussian noise were determined by comparing the PSNR/SSIM differences between synthesized noisy two-photon imaging data and real raw two-photon imaging data. For synthetic images, the image qualities PSNR 20.54 ± 1.54 and SSIM 0.12 ± 0.03 are close to the real raw image qualities PSNR 20.78 ± 0.18 and SSIM 0.14 ± 0.01 (Table 1).

**TABLE 1:** Comparison of denoising performances for the raw two-photon Ca^2+^ imaging dataset in terms of PSNR and SSIM.

Method	PSNR	SSIM	*P* value (model blind spatiotemporal filtering network versus other methods)
Raw	20.78 ± 0.18	0.14 ± 0.01	PSNR: 1.99e-104/SSIM: 2.42e-238
**Model blind spatiotemporal filtering network**	**36.46 ± 1.60**	**0.95 ± 0.01**	N.A.
Spatiotemporal filtering network without model blind learning	35.40 ± 0.79	0.93 ± 0.01	PSNR: 2.17e-15/SSIM: 9.71e-105
DnCNN	33.95 ± 1.01	0.92 ± 0.01	PSNR: 8.70e-53/SSIM: 4.04e-109
BM3D	32.62 ± 0.90	0.87 ± 0.01	PSNR: 7.56e-71/SSIM: 5.88e-106
BM4D	23.69 ± 0.34	0.30 ± 0.02	PSNR: 2.97e-99/SSIM: 1.80e-163
ViDeNN	34.12 ± 0.68	0.94 ± 0.01	PSNR: 5.19e-34/SSIM: 5.24e-51
FITVNet	35.94 ± 1.12	0.93 ± 0.01	PSNR: 0.01/SSIM: 1.56e-48

*Our proposed method is highlighted in bold. Data are presented as mean ± SD. Statistical tests were calculated using the paired *t* test. N.A., not applicable.*

**FIGURE 3:**
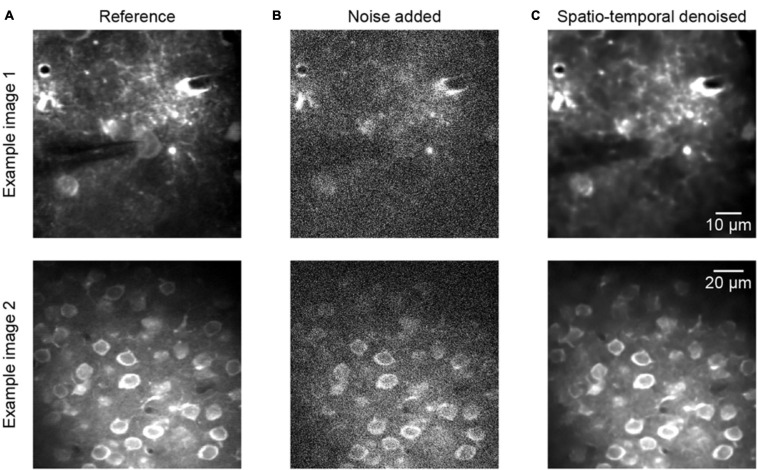
Denoising performance of two representative synthetic two-photon imaging frames. **(A)** The ground truth references. **(B)** Poisson and Gaussian noises were added to the two-photon images: example 1 PSNR 18.56/SSIM 0.07 and example 2 PSNR 17.27/SSIM 0.09. **(C)** Restored images after application of the model blind spatiotemporal filtering method; example 1 PSNR 38.27/SSIM 0.95 and example 2 PSNR 35.94/SSIM 0.90.

Figure 3 shows two representative examples of two-photon Ca^2+^ imaging frames at two different scales, each including a reference image, noise added image, and spatiotemporal denoised image. The quantitative comparisons with respect to the PSNR and SSIM values are listed in the caption of Figure 3, and we can see that the image qualities are degraded after adding noise to the reference of clean data (Figures 3A,B, example 1 PSNR 18.56/SSIM 0.07, example 2 PSNR 17.27/SSIM 0.09). However, the quality of the images was improved considerably after the process of spatiotemporal denoising (Figure 3C, example 1 PSNR 38.27/SSIM 0.95, example 2 PSNR 35.94/SSIM 0.90). The denoised images are well restored, and the SSIM values increase from ∼0.1 to ∼0.9. Thus, the proposed method achieved dramatic image quality improvements in the validation tests.

**FIGURE 4:**
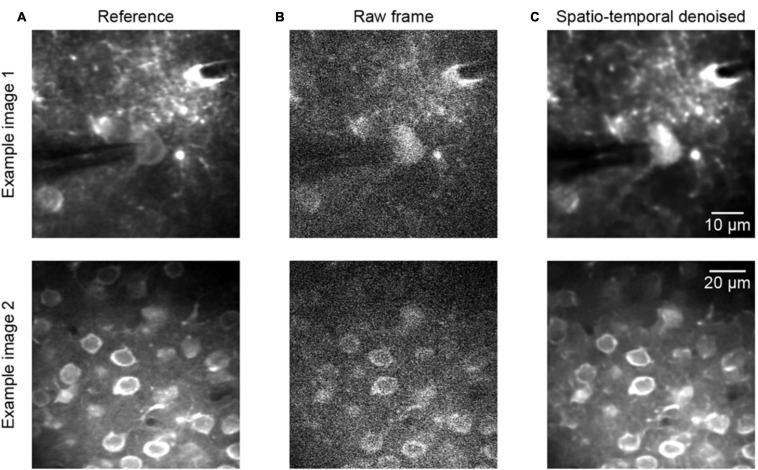
Denoising performance of two representative raw two-photon imaging frames. **(A)** The ground truth references. **(B)** Raw single frames of two-photon imaging data (the middle FOV of the three FOV stack): example 1 PSNR 20.54/SSIM 0.14 and example 2 PSNR 17.85/SSIM 0.11. **(C)** Restored images after application of the model blind spatiotemporal filtering method: example 1 PSNR 32.35/SSIM 0.95 and example 2 PSNR 24.70/SSIM 0.87.

To continue the performance validation of the proposed method, real raw two-photon imaging data were processed and a quantitative assessment was performed. Comparing the experimental validation results in Figures 3, 4, which show the results for the raw two-photon images, it is observed that the impressive performance of the proposed method is replicated. In Figure 4, we can see that the quality of the two representative examples (Figure 4A) is improved; the raw noisy imaging frames (Figure 4B, example 1 PSNR 20.54/SSIM 0.14, example 2 PSNR 17.85/SSIM 0.11) were clearly restored after processing (Figure 4C, example 1 PSNR 32.35/SSIM 0.95, example 2 PSNR 24.70/SSIM 0.87), with the SSIM values increasing from ∼0.1 to ∼0.9. Although we did not define the precise noise model for training with real raw imaging data, the neural network learned the mapping directly from the noisy data, thus exhibiting the ability to denoise acquired two-photon Ca^2+^ imaging data afflicted with complex noises.

**FIGURE 5:**
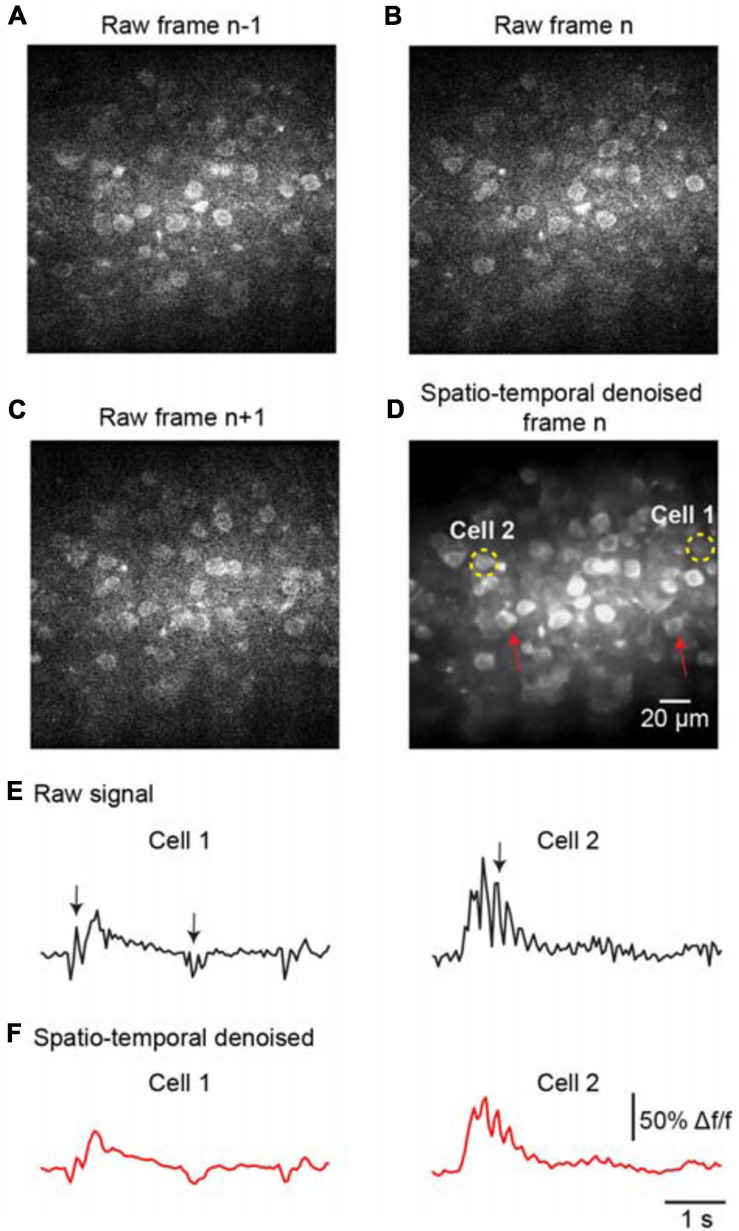
Denoising performance evaluation for temporal inconsistency. A stack of two-photon imaging data: **(A)** frame *n-1*, **(B)** frame *n*, and **(C)** frame *n*+ 1. Restored images using **(D)** the proposed model blind spatiotemporal filtering method: PSNR 31.27/SSIM 0.87. The red arrows indicate the inconsistent areas due to motion changes. The yellow dashed circles indicate the ROIs for the cells. **(E)** The raw Ca^2+^ signals extracted from the cells in **(D)**. **(F)** The extracted Ca^2+^ signals were processed by model blind spatiotemporal filtering.

To further evaluate the denoising performance for handling temporal inconsistencies, we investigated the effectiveness of the proposed method for the imaging data suffering from motion artifacts. As the representative stack of three consecutive imaging frames shown in Figures 5A–C, the temporal inconsistencies were arising from motion-induced nonuniform deformations. For instance, the cells marked by red arrows in Figure 5D were hardly visible in the raw image frame (Figure 5B) due to motion in the imaging data. The proposed spatiotemporal processing method captured temporal information among successive frames and modeled spatial noises and temporal deformations within one network; thus, it successfully restored the imaging data (Figure 5D, PSNR 31.27/SSIM 0.87), particularly for the temporal inconsistent areas in the FOV. In addition, we analyzed the temporal characteristics of the denoised data by extracting Ca^2+^ signals for the imaged cells. The results demonstrate that motion led to spike-like changes in the time series, as indicated by the black arrows in Figure 5E, and these spike-like changes were successfully reduced without harming the real neuronal signals by using our proposed method (Figure 5F).

**FIGURE 6:**
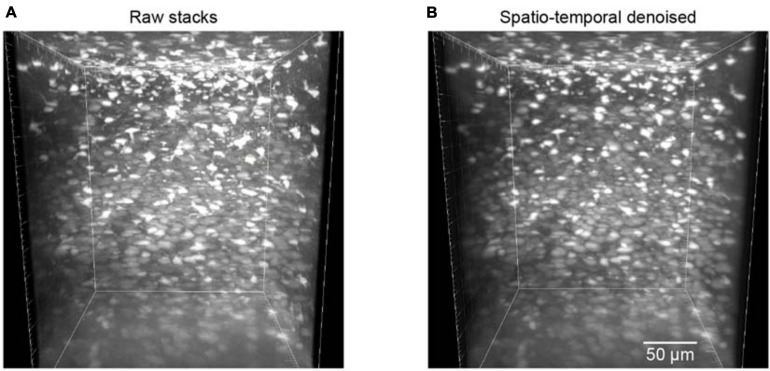
Reconstruction of volumetric imaging data (z-stacks). **(A)** The raw image stacks (BIQI score of 70.67 ± 6.64). **(B)** The model blind spatiotemporal filtered image stacks (BIQI score of 41.28 ± 8.97). Data are presented as mean ± SD.

The trained neural network was also used to test the restoration of raw volumetric two-photon Ca^2+^ imaging data. This testing dataset was composed of 700 raw images for different imaging depths in the mouse cortex. For these volumetric images, as the imaging planes here were not fixed, we could not estimate the ground truth reference by simply averaging many imaging frames. Hence, we applied the BIQI to quantify the image restoration effect for such imaging data. A 3D volumetric imaging data example is shown in Figure 6A; the average BIQI score is 70.67 ± 6.64. After processing *via* the proposed denoising approach, the average BIQI score decreased to 41.28 ± 8.97 (Figure 6B). We can see from the 3D reconstruction that the imaging data quality was clearly improved (*P* = 9.03e-87, paired *t* test) after applying the spatiotemporal filtering process.

### Comparison of Different Methods for Spatial Denoising

**FIGURE 7:**
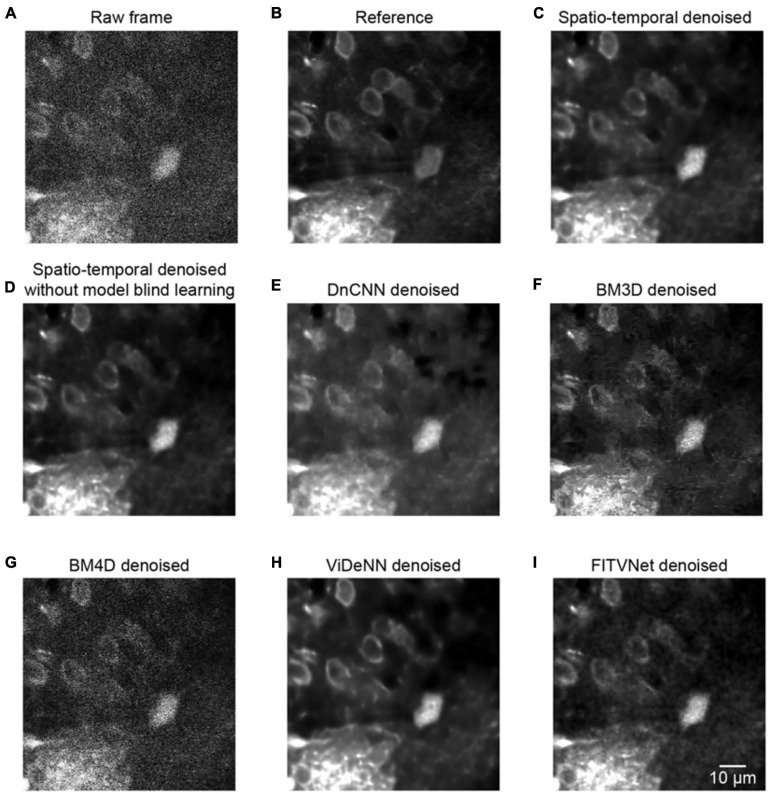
Comparison of denoising performance for seven methods. **(A)** A single raw frame of the two-photon imaging data. **(B)** The ground truth reference. Restored images using **(C)** the proposed model blind spatiotemporal filtering method, **(D)** spatiotemporal filtering network without model blind learning, **(E)** DnCNN method, **(F)** BM3D method, **(G)** BM4D method, **(H)** ViDeNN method, and **(I)** FITVNet method.

To assess the denoising performance for two-photon Ca^2+^ imaging data in greater detail, we compared the proposed method with the current state-of-the-art denoising methods. We tested a dataset composed of 600 imaging frame pairs to evaluate the restoration performance of the networks. We compared our approach with BM3D and DnCNN, which are used for denoising single images, and with the popular video denoising algorithm BM4D (Figure 7 and Table 1). We implemented these algorithms using the default key parameter settings for DnCNN, with the sigma level set to 50 for BM3D, and the noise estimation mode activated for BM4D.

As evident from the testing results presented in Figure 7, our approach provides a result that bears close resemblance to the reference (Figures 7A–C). In addition, as demonstrated by the results listed in Table 1, the model blind spatiotemporal processing exhibits superior results compared with existing denoising tools both in terms of PSNR (36.46 ± 1.60) and SSIM (0.95 ± 0.01). Moreover, we performed the analysis for testing the performance of the spatiotemporal filtering network using only synthesized data, and we obtained good results (Figure 7D and Table 1, spatiotemporal filtering network without model blind learning). It indicates that the Poisson–Gaussian noise model is suitable for simulating synthesized two-photon imaging data. Of the alternative deep learning methods for performing spatial denoising, DnCNN was close to matching the image quality achieved by the proposed method (Figure 7E), ranking well in our analysis. BM3D also achieved relatively strong results but struggled to handle all the noise components in the two-photon Ca^2+^ imaging data (Figure 7F), with the result that the processed image still appears slightly corrupted compared with the reference image. Surprisingly, BM4D performed poorly, with the denoised image retaining a high level of residual noise (Figure 7G) despite the activation of the noise estimation mode. The PSNR and SSIM results show that image quality improvement is limited using the BM4D approach. Here, we speculate that, when the noise is too complex to estimate in the real raw image, BM4D might fail to denoise the images, and thus, noises were still present after denoising.

In addition, we also compared our method with two other spatiotemporal denoising methods: ViDeNN (Claus and Van Gemert, 2019) and FITVNet (Wang C. et al., 2020). As the results (Figures 7H,I and Table 1) demonstrate, both these two spatiotemporal denoising methods performed well in our denoising tests, and their denoising performances are higher than the spatial filtering methods, i.e., DnCNN and BM3D, and are at the same level as the performance of the spatiotemporal filtering network without model blind learning. In comparison with those methods, our proposed model blind spatiotemporal filtering network provided the best denoising results.

### Comparison of Different Methods for Temporal Denoising

**FIGURE 8:**
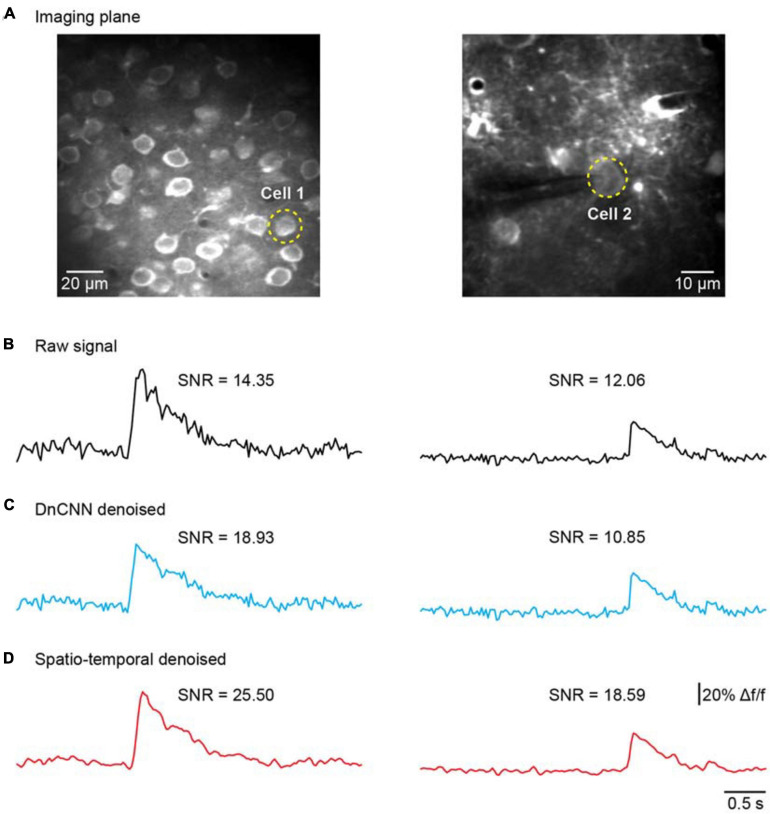
Comparison of temporal denoising performances. **(A)** Two representative imaging planes generated by averaging; the yellow dashed circles indicate the ROIs in the cells. **(B)** The raw Ca^2+^ signals extracted from the cells in **(A)**. **(C)** The extracted Ca^2+^ signals with imaging data processed by the DnCNN. **(D)** The extracted Ca^2+^ signals with imaging data processed by model blind spatiotemporal filtering.

We further addressed the issue of neuronal activity denoising *via* a time series of recorded fluorescence. Ca^2+^ transients were recorded and extracted *in vivo* for individual neurons in the brain (Figure 8A) to quantify the amplitude of Ca^2+^ transients and their SNR values. For these tests, we compared our method with the DnCNN method using a dataset of Ca^2+^ transients (*n* = 20). The spatiotemporal filtered signals showed significantly lower fluctuations and higher SNRs for all Ca^2+^ transients tested (Figures 8B–D), with the average SNR values summarized in Table 2. As we can see in Figures 8B–D, our method preserved the temporal dynamics of Ca^2+^ activity after the spatiotemporal filtering. Compared with raw signals, the spatiotemporal filtered signals demonstrate that the peak amplitudes of the Ca^2+^ transients (the signal part of SNR) were unchanged and the temporal fluctuations (the noise part of SNR) were clearly reduced, and thus, we obtained significantly improved signal quality in temporal domains. Hence, the mean SNR of individual Ca^2+^ transients processed by spatiotemporal filtering (34.87 ± 14.77) was significantly larger than that measured for the raw signals (*P* < 0.001, paired *t* test), which would facilitate the detection of neuronal Ca^2+^ transients. As our method approaches the spatial and temporal denoising problems simultaneously, these results also indicate that model blind spatiotemporal filtering can obtain higher SNR values than the methods that only perform spatial filtering (e.g., DnCNN) of the imaging data, and is therefore more suitable for two-photon Ca^2+^ imaging data restoration. In addition, it is worth to note that using the DnCNN algorithm removed only spatial noise, and it did not consider temporal consistencies in the imaging data. Therefore, using spatial filtering might enlarge the temporal fluctuation of the ROI-based fluorescence and resulted in lower SNR of Ca^2+^ transients than the raw signals.

**TABLE 2:** Comparison of denoising algorithms for the SNR of Ca^2+^ transients.

Method	SNR	*P* value (model blind spatiotemporal filtering network versus other methods)
Raw signal	23.16 ± 9.95	2.12e-8
DnCNN	19.70 ± 7.52	3.37e-4
**Model blind spatiotemporal filtering network**	**34.87 ± 14.77**	N.A.

*Our proposed method is highlighted in bold. Data are presented as mean ± SD. Statistical tests were calculated using the paired *t* test. N.A., not applicable.*

## Discussion

In this work, we used a simple noise-to-noise learning method to conduct fine-tuning on a residual convolutional network, which was trained initially with Poisson and Gaussian noises. In addition, we combined spatial and temporal information to enhance the processing of two-photon imaging Ca^2+^ data and restore the imaging data. The proposed method performed impressively both when processing imaging data with artificial noise and real raw two-photon imaging data. Moreover, we show that our method achieves strong results both for functional imaging data and for morphological imaging data. The testing results demonstrate that both the spatial and temporal SNR of the imaging data are improved significantly using our approach. A comparative analysis of our method for imaging data denoising against previously reported denoising methods highlights the potential of our method as a powerful denoising tool, with our method achieving superior PSNR and SSIM values. This study represents the first step in utilizing model blind spatiotemporal processing for Ca^2+^ imaging data; it can tackle complex noises without prior knowledge of the input data. Therefore, adopting this image restoration process into the neuroimaging data analysis arsenal will simplify downstream analyses, such as motion correction, detection, and segmentation of cells.

We have demonstrated that the neural network can perform spatiotemporal denoising of two-photon Ca^2+^ imaging data directly, achieving remarkable restoration performance (Tables 1, 2). Unlike single-image restoration methods, such as BM3D (Dabov et al., 2007) and DnCNN (Zhang et al., 2017), our proposed approach extracts relevant spatiotemporal information from consecutive frames of imaging data. This data-driven approach proves efficient and flexible for extracting arbitrary features in spatial and time domains. Therefore, as we have demonstrated in our results, it may provide a more robust denoising performance when the imaging data are degraded owing to temporal inconsistencies (e.g., Figure 5), such as object motion and brightness changes. In our work, we trained the network with pairs of image stacks using L2 loss; the network was learning to generate the average of many plausible restored images as prediction, and thus, it resulted in a blurring effect for the output of denoising. Although the spatial blurriness may cause issues in visualization of fine cell features, our analysis results for image quality and neuronal activity show that the spatiotemporal processed imaging data preserve the cell morphology features and temporal dynamics of neuronal activity (Figures 5, 8) well; hence, no important features were lost due to blurring.

Furthermore, as recent studies (Lehtinen et al., 2018) have shown that image restoration can be learned without clean ground truth data, we developed a network learning approach without requiring a precise noise model. Inspired by a recently reported work (Ehret et al., 2019), we trained our network using synthetic noisy imaging data and fine-tuned it using raw imaging data, with the result that it does not require clean data, which simplifies the preparation of the training data. Combining model blind training with transfer learning methods, such as fine-tuning, the network can potentially also achieve high restoration performance and be generalized to process diverse experimental recordings using only a small amount of data. This enables the processing of degraded data acquired from various imaging systems. In addition, it is also worth to note that modeling measurement noise with temporal or spatial structure may be more realistic and be better for the initial training of the network and may facilitate the noise-to-noise learning during fine-tuning stage, which we plan to test in our future work.

Finally, the calculation time for performing the imaging data restoration is fast, which suggests that it is suitable to conduct high-speed real-time processing, which offers an advantage for neurobiology experiments requiring online observations.

## Data Availability Statement

The imaging data and codes supporting the conclusions of this article are available from the corresponding author upon reasonable request.

## Ethics Statement

This study was approved by the Institutional Animal Care and Use Committee of Third Military Medical University. All experimental procedures were conducted in accordance with Animal Ethical Guidelines of the Third Military Medical University Animal Care and Use Committee.

## Author Contributions

HJ, XC, XinL, CZ, and XiaL contributed to the design of the study. JP and MW performed the imaging experiments and acquired the data. LL, YX, and XiaL designed the method. LL, YX, JG, SL, and YL processed the datasets. HJ, XC, XinL, CZ, and XiaL wrote the manuscript with help from all the other authors. All authors contributed to the article and approved the submitted version.

## Conflict of Interest

The authors declare that the research was conducted in the absence of any commercial or financial relationships that could be construed as a potential conflict of interest.
